# Second malignant neoplasms after childhood cancer: A nationwide population-based study in Korea

**DOI:** 10.1371/journal.pone.0207243

**Published:** 2018-11-15

**Authors:** Hee Young Ju, Eun-Kyeong Moon, Jiwon Lim, Byung Kiu Park, Hee Young Shin, Young-Joo Won, Hyeon Jin Park

**Affiliations:** 1 Center for Pediatric Cancer, National Cancer Center, Goyang, Korea; 2 Cancer Survivor Branch, National Cancer Control Institute, National Cancer Center, Goyang, Korea; 3 Cancer Registration and Statistics Branch, National Cancer Center, Goyang, Korea; 4 Department of Pediatrics, Cancer Research Institute, Seoul National University College of Medicine, Seoul, Korea; Mercy Clinic, UNITED STATES

## Abstract

**Background:**

Second malignant neoplasm is one of the most devastating late effects of childhood cancers. This study aimed to evaluate the incidence and survival outcomes of patients developing second malignant neoplasms (SMNs) after surviving childhood cancer in Korea.

**Methods:**

Medical data of childhood cancer patients diagnosed between 1993 and 2012 were obtained from the Korea Central Cancer Registry. The risk of developing SMNs was calculated using standardized incidence ratio (SIR), excess absolute risk (EAR), and cumulative risk. Kaplan-Meier survival curves were estimated, stratified by SMN status.

**Results:**

A total of 28,405 childhood cancer patients were diagnosed in the study period, and 337 (1.2%) developed SMN. The total follow-up period was 197,359 person-years at risk (PYR), with a median follow-up duration of 5.6 years. Overall SIR was 20.0, which was 23.2 in women, and 17.6 in men. The overall EAR was 16.4 per 10,000 PYR. The most common types of SMNs, in order of incidence, were other malignant epithelial neoplasms, leukemia, and soft tissue sarcomas. The cumulative incidence of developing SMNs was 0.7% at 5 years, 1.2% at 10 years, and 2% at 15 years. After primary cancer diagnosis, the 10-year overall survival rate of patients with SMNs was 65.1%, which was lower than the 73.4% in patients without SMN. After SMN diagnosis, the 10-year overall survival rate was 55.8%.

**Conclusion:**

Through this registry-based study of 5.6 years of follow up, childhood cancer survivors were found to be at 20-fold higher risk of developing a malignant neoplasm compared to the general population. The majority of malignant neoplasms are malignant epithelial neoplasms, leukemia, and soft tissue sarcomas. Continued surveillance for assessing long-term risks, and guidance for appropriate long-term follow up of childhood cancer survivors, are needed.

## Introduction

Over the past two decades in Korea, the incidence of childhood cancer has increased substantially. A recent nationwide childhood cancer study in Korea showed that the age-standardized incidence rate for all cancers among children aged 0 to 14 years was 134.9 per million children in 1999–2011 [1]. The age-standardized incidence rate increased from 117.9 per million children in 1999 to 155.3 per million children in 2011, with an annual percentage change of 2.4%. However, the survival rate of childhood cancer has also increased [1], which may be a result of improved treatment and health infrastructure [2].

On the other hand, the increase of childhood cancer survivors has resulted in a substantial increase in the incidence of second malignant neoplasms (SMNs) after childhood cancer [3]. The occurrence of SMNs in childhood cancer survivors reduces their life expectancy and negatively impacts their quality of life [4]. Overall, childhood cancer survivors have a longer life expectancy than adult cancer survivors, and SMN and secondary chronic health problems across the life span will influence the patient, the patient’s family, and society [5]. Second neoplasms have been known to be major contributors to excess total cumulative burden of chronic health conditions [6]. SMN has also been reported to be a significant factor related to financial or other hardships experienced by childhood cancer survivors [7].

A proper surveillance program for childhood cancer patients is needed to detect other malignant diseases and provide more preemptive management. Research has consistently demonstrated that the incidence of neoplasms in patients who have previously had cancer is higher than that in the general population [4, 8–11]. Early detection and appropriate management of these patients would influence their total survival rate.

According to previous studies, it has been reported that risk of SMN increases with follow-up time, and that cancer treatment determines the risk of secondary cancer [12–15]. The risk of developing SMNs is unequal between different types of cancer [11, 12]. Possible reasons for this difference have been explained as follows: difference in onset age of primary cancer; prevalence of secondary cancer risk factors, including primary cancer treatments; environmental exposures; and genetic susceptibility [3]. Further study of SMN incidence would help clarify characteristics of the group at highest risk for developing SMNs, as well as elucidate the proper surveillance methods for at-risk patients and a better understanding of the time period in which SMNs develop.

The total number of childhood cancer survivors in Korea is estimated to be approximately 30,000; however, recent statistics for SMNs in Korea are not yet available. A previous retrospective multicenter study involving 11 centers in Korea reported that 102 cases of second malignant neoplasm after childhood cancer were treated between 1998 and 2011 [16]. However, there has been no nationwide study about SMN after childhood cancer in Korea.

Thus, in the present study, we aimed to evaluate the incidence and survival outcomes of SMNs that occurred in childhood cancer survivors in Korea, through analysis of national registry data.

## Materials and methods

### Data sources

Registry-based data on childhood cancer patients (aged 0–19 years old) diagnosed between 1993 and 2012 were obtained from the Korea National Cancer Incidence Database (KNCIDB) of the Korea Central Cancer Registry (KCCR). The KCCR is a population-based cancer registry that was begun in 1980 by the Korean Ministry of Health and Welfare, and which has covered the entire Korean population since 1999. The KNCIDB includes information about sex, age, date of first diagnosis, primary tumor site, and method of diagnosis. Detailed information about KCCR data has been reported previously [17]. Mortality data was earned from the cancer mortality database of the Statistics Korea.

We considered that primary malignancy treatment did not affect a second cancer diagnosis that was established within 6 months of the first cancer diagnosis. Therefore, patients who developed a second malignancy within the first 6 months of follow-up were excluded from the study [18]. As a result, a total of 28,405 patients were analyzed with childhood cancer between 1993 and 2012. The institutional review board at the National Cancer Center approved this study (NCC2015-0234).

### Statistical methods

Childhood cancer patients in this study were classified according to the diagnosis of the SMN, using the 12 main diagnostic groups of the International Classification of Childhood Cancers, 3^rd^ edition [19]. SMNs included all observed cases of subsequent cancer, in accordance with the Surveillance, Epidemiology, and End Results Program (SEER) [20]. The expected number of cancers was estimated by multiplying the number of person-years at risk (PYR) by age, sex, and calendar period-specific cancer incidence rates in the general population. As a measure of risk for developing SMNs, standardized incidence ratios (SIRs) were calculated as the observed number of SMNs divided by the expected number of malignant neoplasms in the general population. The 95% confidence intervals (CIs) were calculated assuming the Poisson distribution for the observed cases. In this study, the mid-year population, obtained from the Statistics Korea (http://kosis.kr) was used as the general reference population. To measure the overall burden of SMN, excess absolute risks (EARs) of SMN were calculated by dividing the difference between the observed number and the expected number by the PYR, and were presented as numbers per 10,000 PYR. The number of PYR was defined from 6 months after the date of the first childhood cancer diagnosis to the date of death or the end of this study, whichever occurred first. In addition, we calculated the cumulative incidence of second primary cancer, which was defined from the time of first cancer diagnosis.

Kaplan-Meier survival analyses were done for primary cancer patients and SMN patients. Survival differences between primary cancer patients and SMN patients were estimated using a log-rank test. All statistical tests were two-sided and considered statistically significant for *P*-values <0.05. All calculations of SIR and EAR were performed using the SEER*Stat software (version 8.1.5, National Cancer Institute, Bethesda, MD). The cumulative incidence rates and survival curves were generated, and log-rank tests were performed in SAS (version 9.2, SAS Institute, Cary, NC) and Stata (http://www.stata.com/).

## Results

Malignant neoplasm was diagnosed in 28,405 patients aged 0–19 years, in 15,452 men and 12,953 women, who contributed a total of 197,359 PYR over the 20-year study period (1993–2012). Among these patients, 340 cases of subsequent malignant neoplasms were observed in 337 patients, including 3 patients who developed third cancers (Table 1).

**Table 1:** Summary statistics of childhood cancer patients (0–19 years old).

Characteristic	First malignant patients (n = 28,405)	Subsequent malignant neoplasm (n = 337)^†^
	Number (%)	Number (%)
Sex		
Male	15,452 (54.4)	169 (50.1)
Female	12,953 (45.6)	168 (49.9)
Age at primary diagnosis (year)		
<1	2,075 (7.3)	21 (6.2)
1–4	6,240 (22.0)	63 (18.7)
5–9	4,836 (17.0)	56 (16.6)
10–14	6,210 (21.9)	79 (23.4)
15–19	9,044 (31.8)	118 (35.0)
Time period of diagnosis		
1993–1997	6,523 (23.0)	97 (28.8)
1998–2002	7,311 (25.7)	105 (31.2)
2003–2007	7,314 (25.7)	103 (30.6)
2008–2012	7,257 (25.5)	32 (9.5)
Total person-years of follow-up	197,359	3039.5
Median follow-up, years (range)	5.6 (0–19.5)	8.1 (0–19.5) ^‡^
Median age at initial diagnosis, years (range)	11 (0–19)	12 (0–19)
Median age at secondary diagnosis, years (range)	-	16 (1–36)
Cases according to diagnosis (according to ICCC-3)*		
I. Leukemias, myeloproliferative diseases, and myelodysplastic diseases	8,226 (29.0)	61 (17.9)
II. Lymphomas and reticuloendothelial neoplasms	3,237 (11.4)	6 (1.8)
III. CNS and miscellaneous intracranial and intraspinal neoplasms	3,361 (11.8)	30 (8.8)
IV. Neuroblastoma and other peripheral nervous cell tumors	1,320 (4.6)	8 (2.4)
V. Retinoblastoma	639 (2.2)	-
VI. Renal tumors	817 (2.9)	5 (1.5)
VII. Hepatic tumors	492 (1.7)	4 (1.2)
VIII. Malignant bone tumors	2,106 (7.4)	31 (9.1)
IX. Soft tissue and other extraosseous sarcomas	1,554 (5.5)	46 (13.5)
X. Germ cell tumors, trophoblastic tumors, and neoplasms of gonads	2,806 (9.9)	12 (3.5)
XI. Other malignant epithelial neoplasms and malignant melanomas	3,263 (11.5)	111 (32.6)
XII. Other and unspecified malignant neoplasms	584 (2.1)	26 (7.6)

^†^ Subsequent malignant neoplasms included 340 subsequent (second and later) cancer cases in 337 patients. Data about sex, age, diagnosed time, follow up period and median age was gathered from the data of 337 patients, but diagnoses include 340 cases.

^‡^ Median follow-up after the date of second malignant neoplasms diagnosis.

* Number of cases by cancer diagnosis. (Total number 340, percentage expressed by cases)

The median age at initial diagnosis was 11 years old and the median follow-up period was 5.6 years. The most common type of SMN was ‘other malignant epithelial neoplasms and malignant melanomas’ (Group XI), diagnosed in 111 cases. This group is composed of adrenocortical carcinomas, thyroid carcinomas, nasopharyngeal carcinomas, malignant melanomas, skin carcinomas, and other and unspecified carcinomas. Thyroid carcinoma (51 cases, 45.9%) was the most common type of neoplasm in this group (Table 2).

**Table 2:** Subsequent malignant neoplasms, standardized incidence ratios, and excess absolute risks.

Diagnostic group of subsequent malignant neoplasms	Total				
	Obs	Exp	SIR	95% CI	EAR
All cancers	340	17.0	20.0*	18.0–22.3	16.4
I. Leukemias, myeloproliferative diseases, and myelodysplastic diseases	61	4.6	13.2*	10.1–16.9	2.9
II. Lymphomas and reticuloendothelial neoplasms	6	2.3	2.7	1.0–5.8	0.2
III. CNS and miscellaneous intracranial and intraspinal neoplasms	30	2.0	14.8*	10–21.2	1.4
IV. Neuroblastoma and other peripheral nervous cell tumors	8	0.4	18.4*	8.0–36.3	0.4
V. Retinoblastoma	0	0.2	-	-	-
VI. Renal tumors	5	0.3	16.3*	5.3–38.0	0.2
VII. Hepatic tumors	4	0.2	18.0*	4.9–46.0	0.2
VIII. Malignant bone tumors	31	1.4	22.7*	15.4–32.2	1.5
IX. Soft tissue and other extraosseous sarcomas	46	1.0	44.8*	32.8–59.7	2.3
X. Germ cell tumors, trophoblastic tumors, and neoplasms of gonads	12	1.7	7.1*	3.7–12.5	0.5
XI. Other malignant epithelial neoplasms and malignant melanomas	111	2.6	43.0*	35.3–51.7	5.5
XI.b. Thyroid carcinomas	51	1.7	30.8*	23.0–40.5	2.5
XI.f.1. Carcinomas of salivary glands	6	0.1	51.4*	18.9–111.9	0.3
XI.f.2. Carcinomas of colon and rectum	10	0.2	66.6*	31.9–122.4	0.5
XI.f.6. Carcinomas of breast	12	0.04	271.5*	140.3–474.3	0.6
XII. Other and unspecified malignant neoplasms	26	0.3	94.5*	61.7–138.5	1.3

SIR: standardized incidence ratio, CI: confidence interval, EAR: excess absolute risk SIRs were not estimated for 0 observed incidence cases. EAR with a value lower than 0.1 is not shown.

*p<0.05, Excess absolute risk (EAR) is per 10,000 person-years

Following ‘other malignant epithelial neoplasms and malignant melanomas’, the second, third, fourth, and fifth most commonly-observed types of SMNs were ‘leukemias, myeloproliferative diseases, and myelodysplastic diseases’ (Group I, 61 cases), ‘soft tissue and other extraosseous sarcomas’ (Group IX, 46 cases), ‘malignant bone tumors’ (Group VIII, 31 cases), and ‘central nervous system (CNS) and miscellaneous intracranial and intraspinal neoplasms’ (Group III, 30 cases), respectively. The overall SIR was 20.0 (Table 2). The following group of cancers showed a high SIR as SMNs: other and unspecified malignant neoplasms’ (94.5), ‘soft tissue and other extraosseous sarcomas’ (44.8), and ‘other malignant epithelial neoplasms and malignant melanomas’ (43.0).

**Fig 1:**
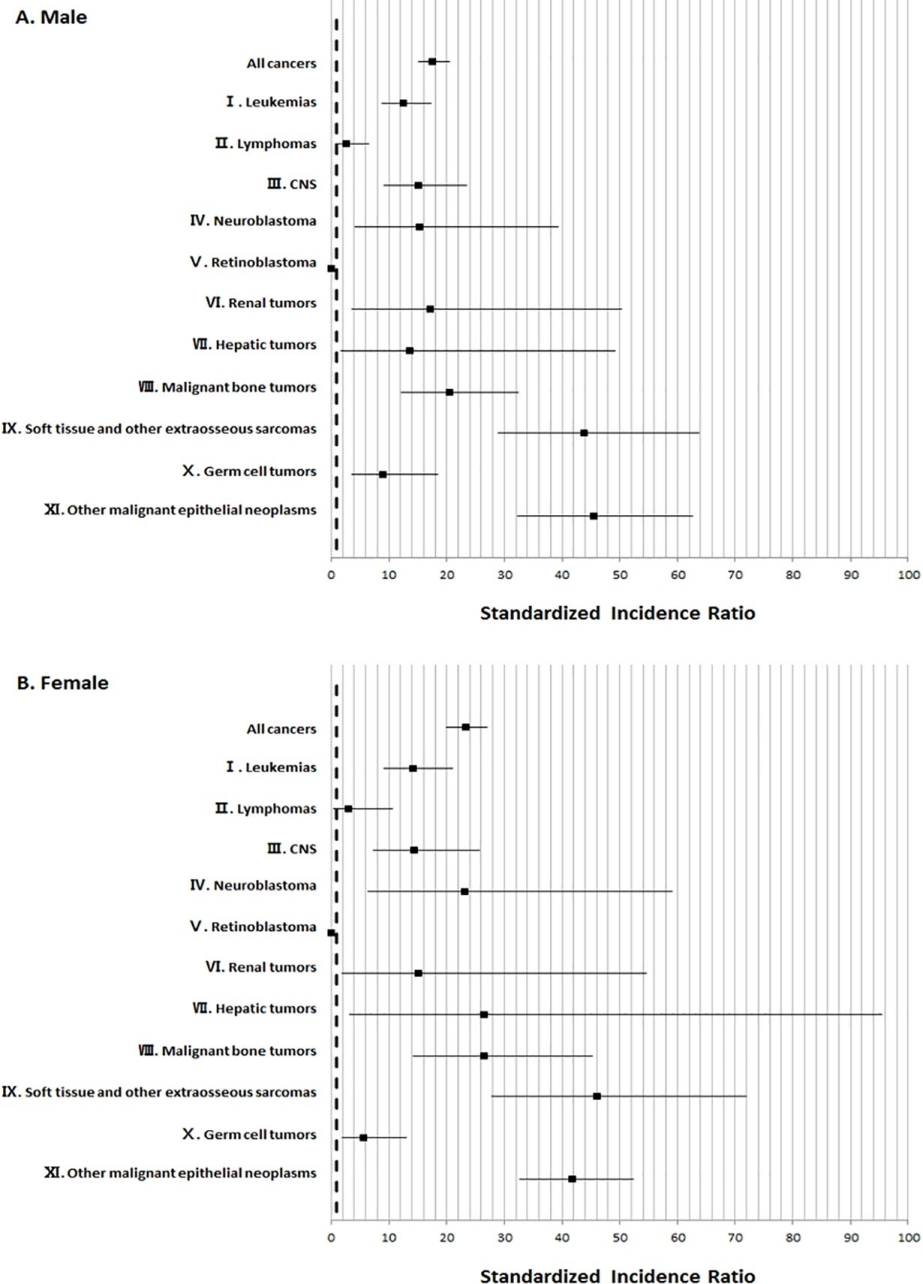
SIRs by sex of SMNs among childhood survivors of initial cancer during 1993–2012. SIR: standardized incidence ratio, EAR: excess absolute risk.

The SIR was higher in women (SIR = 23.3; 95% CI, 19.9–27.1) compared to men (SIR = 17.6; 95% CI, 15.1–20.4), but the difference was not statistically significant (Fig 1).

According to the primary malignant neoplasm types, ‘soft tissue and other extraosseous sarcomas’ showed highest SIR (51.61) for developing SMN. (Table 3). Patients who had soft tissue and extraosseous sarcoma as primary cancer often developed “soft tissue and other extraosseous sarcomas” as secondary cancer (10 of 46 SMNs), followed by “leukemias, myeloproliferative diseases, and myelodysplastic diseases” (9 of 46 SMNs).

**Table 3:** SMN occurrence according to primary cancer type.

Diagnostic group of primary malignant neoplasms	SMN				
	Obs	Exp	SIR	95% CI	EAR
I. Leukemias, myeloproliferative diseases, and myelodysplastic diseases	39	5.2	7.5	5.3–10.2	6.4
II. Lymphomas and reticuloendothelial neoplasms	41	1.9	22.0	15.8–29.9	17.2
III. CNS and miscellaneous intracranial and intraspinal neoplasms	32	1.8	17.8	12.1–25.1	14.9
IV. Neuroblastoma and other peripheral nervous cell tumors	32	1.0	33.1	22.6–46.7	38.6
V. Retinoblastoma	5	0.7	6.8	2.2–15.8	7.2
VI. Renal tumors	8	0.8	10.2	4.4–20.0	10.3
VII. Hepatic tumors	5	0.4	0.4	4.4–31.4	13.6
VIII. Malignant bone tumors	44	1.0	45.2	32.8–60.6	31.3
IX. Soft tissue and other extraosseous sarcomas	46	0.9	51.6	37.8–68.8	43.0
X. Germ cell tumors, trophoblastic tumors, and neoplasms of gonads	43	1.8	23.8	17.2–32.1	17.5
XI. Other malignant epithelial neoplasms and malignant melanomas	35	1.1	30.8	21.4–42.8	14.0
XI.b. Thyroid carcinomas	17	0.6	27.0	15.7–43.2	10.8
XI.f.2. Carcinomas of colon and rectum	3	0.03	86.3	17.8–252.2	36.2
XI.f.6. Carcinomas of breast	3	0.02	186.8	38.5–545.9	50.8
XII. Other and unspecified malignant neoplasms	11	0.3	34.0	17.0–60.1	19.7

SIR: standardized incidence ratio, CI: confidence interval, EAR: excess absolute risk

On comparing between the incidence of primary and secondary cancers types, all 5 retinoblastoma patients with SMN developed secondary “malignant bone tumors”. In addition, “leukemias, myeloproliferative diseases, and myelodysplastic diseases” was the most common type of SMN after primary “malignant bone tumor” (18 of 44 cases, 40.9%) (the absolute value of SMN cases after each cancer was not indicated because of small number of cases).

Almost half (47.4%, n = 161) of SMNs occurred within 5 years of diagnosis of the primary cancer, while 93 cases (27.4%) occurred 5–10 years after diagnosis of primary cancer, and 86 cases (25.3%) occurred more than 10 years after diagnosis of primary cancer (Table 4). According to the latency period after diagnosis of primary cancer, the overall SIR was highest after 10 years from initial cancer diagnosis (SIR = 32.7).

**Table 4:** Standardized incidence ratios and excess absolute risk of second malignant neoplasms in childhood by latent periods, median latency and median age.

Second malignant neoplasms	Latency period (months)												Median latency (years)^†^	Median age^§^
	6–11 months			12–59 months			60–119 months			≥120 months				
	Obs	SIR	EAR	Obs	SIR	EAR	Obs	SIR	EAR	Obs	SIR	EAR		
All cancers	46	27.3*	33.1	115	14.3*	13.7	93	20.2*	14.2	86	32.7*	19.2	5.3	16
I. Leukemias, myeloproliferative diseases, and myelodysplastic diseases	2	4.1	1.1	43	18.1*	5.2	11	9.0*	1.6	5	9.1*	1.0	3	15
II. Lymphomas and reticuloendothelial neoplasms	3	15.3*	2.1	2	2	0.1	1	1.5	0.1	-	-	-	1.3	11
III. CNS and miscellaneous intracranial and intraspinal neoplasms	4	20.3*	2.8	11	11.1*	1.3	7	11.9*	1.0	8	32.7*	1.8	5.4	16
IV. Neuroblastoma and other peripheral nervous cell tumors	3	36.5*	2.2	4	14.1*	0.5	-	-	-	1	63.4*	0.2	1.3	8.5
V. Retinoblastoma	-	-	-	-	-	-	-	-	-	-	-	-	-	-
VI. Renal tumors	2	41.1*	1.5	1	5.4	0.1	2	39.1*	0.3	-	-	-	2.7	20
VII. Hepatic tumors	-	-	-	2	16.2*	0.2	-	-	-	2	96.1*	0.5	8.9	25
VIII. Malignant bone tumors	2	18.1*	1.4	8	13.9*	1.0	17	40.0*	2.7	4	15.8*	0.9	5.6	12
IX. Soft tissue and other extraosseous sarcomas	11	115.9*	8.1	18	39.2*	2.2	7	24.4*	1.1	10	53.9*	2.3	1.9	16
X. Germ cell tumors, trophoblastic tumors, and neoplasms of gonads	3	19.5*	2.1	3	4.1	0.3	3	6.2*	0.4	3	9.7*	0.6	5.0	20
XI. Other malignant epithelial neoplasms and malignant melanomas	6	29.4*	4.3	16	15.3*	1.9	40	55.6*	6.3	49	80.2*	11.1	9.4	22.5
XI.b. Thyroid carcinomas	1	7.9	0.7	6	9.1*	0.7	18	39.6*	2.8	26	63.2*	5.9	10.0	16
XI.f.1. Carcinomas of salivary glands	1	122.8*	0.7	-	-	-	3	81.6*	0.5	2	75.8*	0.5	9.2	16.5
XI.f.2. Carcinomas of colon and rectum	2	160.5*	1.5	1	16	0.1	2	48.3*	0.3	5	147.5*	1.1	6.3	17
XI.f.6. Carcinomas of breast	-	-	-	1	55.3*	0.1	5	424.6*	0.8	6	556.9*	1.4	11.9	20
XII. Other and unspecified malignant neoplasms	10	279.2*	7.4	7	48.5*	0.9	5	82.4*	0.8	4	116.8*	0.9	1.6	10

SIR: standardized incidence ratio, SIRs were not estimated for 0 observed incidence cases. EAR: excess absolute risk (per 10,000 PYR)

*p<0.05

^**†**^Median latency at the year of second malignant neoplasm diagnosis since the year of initial cancer diagnosis.

^**§**^Median age at diagnosis of a second malignant neoplasm.

The overall EAR was 16.4 per 10,000 PYR (Table 2). EAR was the highest in the “other malignant epithelial neoplasms and malignant melanomas” group (5.5). When EAR was analyzed based on time of SMN diagnosis, the “soft tissue and other extraosseous sarcomas” group had the highest EAR of 8.1 per 10,000 PYR at 6–11 months after primary cancer diagnosis. At 12–59 months after primary cancer diagnosis, leukemias had the highest EAR of 5.2 per 10,000 PYR. The “other malignant epithelial neoplasms and malignant melanomas” group had the highest EAR with 6.3 and 11.1 per 10,000 PYR at 60–119 and ≥120 months after the primary cancer diagnosis, respectively. This suggests that a greater increase in incidence corresponds with a longer time period between primary and SMN diagnosis.

**Fig 2:**
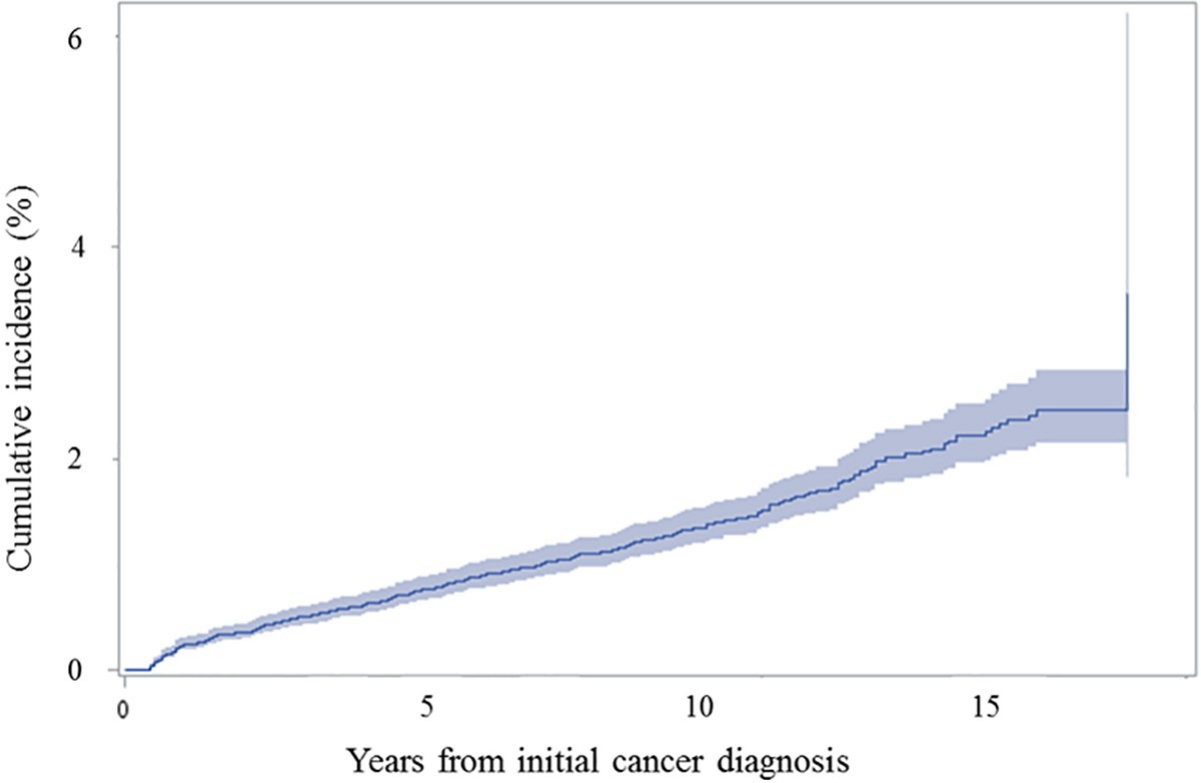
Cumulative incidence of second malignant neoplasms.

The median latency between first and second cancers was 5.3 years, which was shortest for secondary ‘lymphomas and reticuloendothelial neoplasms’ (1.3 years) and ‘neuroblastoma and other peripheral nervous cell tumors’ (1.3 years). On the contrary, ‘carcinomas of breast’ (11.9 years) and ‘thyroid carcinomas’ (10 years) had the longest median latency. The median age at SMN was 16 years, while the development of hepatic tumors occurred at the oldest age (25 years) (Table 4). The cumulative incidence of SMNs was 0.7% at 5 years, 1.2% at 10 years, and 2.0% at 15 years (Fig 2).

**Fig 3:**
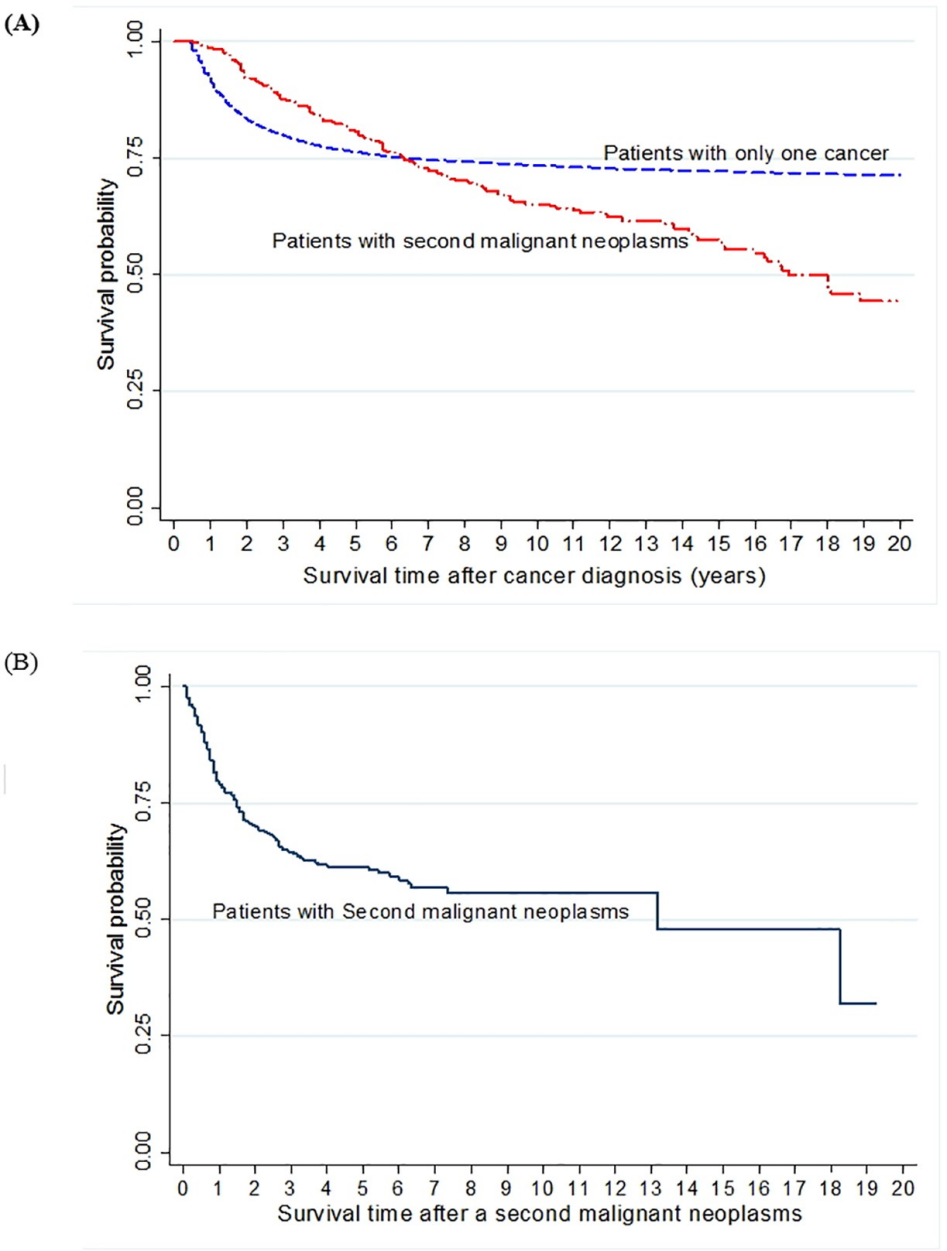
Survival probability of childhood cancer patients. (A) Survival probability of childhood patients with only one primary cancer and second malignant neoplasms. (B) Survival probability of childhood patients after second malignant neoplasms diagnosis.

The 5-year overall survival rates were 76.2% for patients with only one primary cancer and 80.5% for patients with second malignant neoplasms. The 10-year overall survival rate of patients with SMN was 65.1%, lower than the 73.4% overall survival rate of patients without SMN, but statistically non-significant (Fig 3A). The 5-year and 10-year overall survival rates from the onset of the SMNs were 61.2% and 55.8%, respectively (Fig 3B). In particular, among patients with SMN, the 5-year survival rate was highest (87.6%) for those whose SMN was categorized as ‘other malignant epithelial neoplasm and malignant melanoma’, and lowest (34.1%) for those diagnosed with leukemia. In addition, 5-year survival rates of common SMNs were 44.6% (soft tissue and other extraosseous sarcomas), 49.3% (malignant bone tumors), and 35.2% (CNS and miscellaneous intracranial and intraspinal neoplasms) (data not shown).

## Discussion

Through this study, we concluded that risk of SMN in childhood was 20-fold greater in childhood cancer survivors than the risk of developing a malignant neoplasm in the general population. SIR and EAR of SMN among the childhood cancer survivors in this cohort were 20.0 and 16.4, respectively. When compared to previous reports from various nations, SIR in this study was higher than in others, which reported values between 5.0–8.0 [4, 11, 21–23]. This difference may be associated with the shorter follow up period in our study, as SIR is known to be highest during the first 5–9 years and decreases with longer duration of follow up [11, 21]. This can be explained by the low incidence of cancer in the general population aged 0–19 years old, and the relatively much higher incidence of SMN in cancer survivors. Cumulative incidence of SMN after childhood cancer in this study was 1.2% at 10 years and 2.0% at 15 years, which is similar to previous studies. One Japanese study reported 1.1% and 2.6% of CIR at 10 years and 20 years after childhood cancer, respectively [24]. Data from the Childhood Cancer Survivor Study (CCSS) showed the 20-year and 30-year cumulative incidence of SMN to be 3.2% and 9.3%, respectively [22], while a recent U.S. study reported that the cumulative incidence of new subsequent neoplasm and SMNs occurring after age 40 was 34.6% and 16.3%, respectively [14]. Longer follow up is needed to measure the cumulative incidence of SMN in Korean childhood cancer survivors.

When divided according to primary malignant neoplasm, breast cancer showed a particularly high SIR in this study. A high proportion of breast cancer cases occurred after a diagnosis of malignant bone tumor, soft tissue sarcoma, germ cell tumor, or other malignant epithelial neoplasm (ICCC-3 group XI). The median age at diagnosis of breast cancer in Korean women is approximately 10 years younger than that of women in the United States, and this difference may be associated with different environmental and genetic backgrounds between ethnicities [25]. In addition to possible genetic or environmental factors, treatments for sarcomas, germ cell tumors, and other malignant epithelial neoplasms may have influenced the early occurrence of breast cancer in this study. A recent study has shown that the prevalence of BRCA mutations in Korean familial breast cancer patients is similar to that among Western cohorts, but more studies are needed before a concrete conclusion can be drawn [26].

In our study, the risk of SMN was higher in females than in males, concordant with previous studies [4, 8, 11, 22]. Females demonstrated higher SIR for SMNs in the ‘other malignant neoplasm’ group, which includes thyroid carcinomas and carcinomas of the breast, and in the ‘soft tissue sarcoma’ group. The higher risk of SMNs in female survivors of childhood cancer may in part be influenced by breast cancer risk.

In this study, the primary cancer with highest SMN risk was soft tissue sarcoma and malignant bone tumor. Due to lack of data about treatment in our data set, it is impossible to identify the relationship between the treatment and results in SMN. However, previous studies have shown a possible relationship between treatment and SMNs in sarcoma patients. The high incidence of SMN in these groups could be partially related to treatment, including alkylators, anthracyclines, or epipodophyllotoxins [27]. Radiation therapy is also known to increase risk of secondary sarcoma development in previous sarcoma patients [28]. Therefore, efforts to modify treatment in low-risk sarcoma patients could lower the risk of SMN in sarcoma survivors. A second possible reason for the high SMN rate in soft tissue sarcoma and malignant bone tumors may be associated with cancer-prone mutations; for example, *TP53* mutation or *RB1* mutation in osteosarcoma. Newly discovered mutations have been reported in various types of sarcomas [29]. Expansion of knowledge about these mutations will provide more information of pathophysiology and tumorigenesis of SMNs. Genetic study is not routinely done in childhood cancer patients in Korea, but future extended trials of genetic study will provide information not only for cancer treatment but also for SMN surveillance and family management.

Several studies showed high incidence of SMN after treatment of Hodgkin lymphoma [21, 22]. However, in this study, we could not find higher tendency of SMN in lymphoma, possibly because we did not divide the diagnosis of lymphoma according to subtypes. Known common SMNs after childhood Hodgkin Lymphoma are breast cancer, thyroid cancer, or sarcoma, and as latency of these SMNs are long, these could not be detected in the short follow up duration of our study.

According to this study, secondary leukemia or lymphoma mostly occurred within 5 years of the primary diagnosis, and carcinoma of thyroid or breast was found after 5 years of latency. Based on these results, a routine blood test is needed for at least 5 years after cancer treatment to screen for secondary hematologic malignancy. Beyond 5 years, surveillance of thyroid and breast cancers with physical examinations or imaging studies would be beneficial. In short, our results indicate the need of clinical follow up for more than 5 years and emphasizing continued surveillance to patients is of particular importance. As many cancer survivors do not realize the health risks that are related to their childhood cancer, many do not undergo the regular medical follow-up that is recommended for secondary cancer screening [30].

In this study, the 10-year overall survival rate of patients with and without SMN were 65.1% and 73.4%, respectively. Although there are several long-term studies of childhood cancer survivors, only a few studies reported the overall survival of patients who developed SMN after childhood cancer. A recent Japanese study showed overall survival of survivors who developed secondary cancer, before and after 5 years from primary cancer diagnosis, to be 30% and 62%, respectively [24].

This study has several limitations. First, as data was extracted from a population-based cancer registry database, it lacks detailed information about individual treatment methods, such as radiotherapy, chemotherapy and surgical therapy. Several studies have reported that secondary cancer risk is increased by the treatment received for the first cancer [15, 21], with a particular emphasis on the effects of radiotherapy [31, 32]. Hematopoietic stem cell transplantation is also known to increase the risk of SMN, due to the conditioning regimen (with or without total body irradiation), graft-versus-host disease, and post-transplant lymphoproliferative disorder [33–35]. Further study with treatment data is needed to provide recommendations for cancer treatment that consider the risk of SMN. Second, the follow up period of this study was shorter than in previous studies [4, 10, 11, 21], although comparable with some national studies [8, 11]. Several previous studies have shown that breast, thyroid, bone tumor, colorectal, and lung cancer risk increases after Hodgkin disease, and average time to second neoplasm has been reported to be 14.6 years [4, 36]. As follow up time was shorter in the current study, the risk of Hodgkin lymphoma in this study could have been underestimated, and longer follow up could provide different data than reported in this study. Further study and long-term follow up of childhood cancer survivors is needed for more detailed and reliable data. Lastly, this study included only a small number of cases in some diagnostic groups; the study should thus be interpreted with caution. Particularly, caution is required when interpreting SIR values for a small number of cases because these values could be statistically unstable.

In spite of these limitations, this study retains its significance, as many studies concerning SMNs in childhood cancer do not provide the survival data. Furthermore, this study is valuable as the first Korean, nationwide, population-based study of SMN after childhood cancer. This study will be helpful for planning cancer control program strategies such as screening, surveillance, and treatment to decrease the incidence of SMNs and improve childhood SMN survival rates in Korea.

## Conclusion

Our findings suggest that among childhood cancer survivors, the risk of SMN is increased 20-fold when compared with the risk for the general population. Due to this increased risk, continued surveillance of SMN is recommended for childhood cancer survivors. Additionally, attempts should be made to reduce treatment intensity, and to select proper treatment modalities. Further studies with longer follow up time periods are needed to clarify the pattern of SMN, to reveal the etiology and risk factors for SMN in childhood primary-cancer survivors, and to provide an advanced follow up guide for childhood cancer survivors.
